# Molecular beacon-based real-time PCR detection of primary isolates of *Salmonella *Typhimurium and *Salmonella *Enteritidis in environmental and clinical samples

**DOI:** 10.1186/1471-2180-9-97

**Published:** 2009-05-19

**Authors:** Andreas V Hadjinicolaou, Victoria L Demetriou, Maria A Emmanuel, Charalambos K Kakoyiannis, Leondios G Kostrikis

**Affiliations:** 1 Department of Biological Sciences, University of Cyprus, Nicosia, Cyprus; 2 Department of Veterinary Services, Ministry of Agriculture, Natural Resources and Environment, Nicosia, Cyprus

## Abstract

**Background:**

A fast and simple two-step multiplex real-time PCR assay has been developed to replace the traditional, laborious Salmonella serotyping procedure. Molecular beacons were incorporated into the assay as probes for target DNA. Target sequences were regions of the invA, prot6E and fliC genes specific for Salmonella spp. Salmonella Enteritidis and Salmonella Typhimurium, respectively, the two most clinically relevant serotypes. An internal amplification positive control was included in the experiment to ensure the optimal functioning of the PCR and detect possible PCR inhibition. Three sets of primers were used for the amplification of the target sequences. The results were compared to those of the Kauffmann-White antigenic classification scheme.

**Results:**

The assay was 100% sensitive and specific, correctly identifying all 44 Salmonella strains, all 21 samples of S. Enteritidis and all 17 samples of S. Typhimurium tested in this work. Therefore, the entire experiment had specificity and sensitivity of 100%. The detection limit was down to 10 copies of DNA target per 25 μl reaction.

**Conclusion:**

The assay can amplify and analyse a large number of samples in approximately 8 hours, compared to the 4 to 5 days conventional identification takes, and is thus considered a very promising method for detecting the two major serotypes of *Salmonella *quickly and accurately from clinical and environmental samples.

## Background

*Salmonella *is a gram-negative, facultative anaerobic, flagellated bacterium. It is the pathogenic agent of salmonellosis, a major cause of enteric illness and typhoid fever, leading to many hospitalisations and a few rare deaths if no antibiotics are administered. *Salmonella *outbreaks are linked to unhygienic food preparation, cooking, reheating and storage practices. The bacterium can be isolated from raw meat and poultry products as well as from milk and milk-based products [1]. The detection of *Salmonella *therefore remains a highly important issue in microbiological analysis for food safety and standards.

Because the nomenclature for the *Salmonella *genus is at times confusing, this publication will follow the current literature [2,3]. The CDC [3] distinguishes two *Salmonella *species (or subgenera): *S. enterica and S. bongori. S. enterica *is further divided into six subspecies, of which *S. enterica *subsp. *enterica *is the most clinically significant, causing 99% of *Salmonella *infections. In the present study we are concerned with its two main serovars: *Salmonella enterica *serovar Typhimurium (group D) denoted *S*. Typhimurium, and *Salmonella enterica *serovar Enteritidis (group B) denoted *S*. Enteritidis, which are the most commonly isolated *Salmonellae *from food-borne outbreaks.

Identification of the disease-causing *Salmonella *serovars is currently a lengthy process, and its initial isolation from food samples can be difficult as the bacteria can be present in small numbers and many closely related bacteria may be found within the same sample [4]. For this reason, pre-enrichment steps are required for all samples [5,6]. The current accepted method for isolation of *Salmonella *from foodstuffs is a well established procedure – ISO 6579, laborious and time-consuming, taking up to 5 days to complete [7,8]. The most widely-used method used to characterise *Salmonella *into its subspecies is the Kauffman-White serotyping system [9], based on the variability of the O, H and Vi antigens [9-11]. Apart from being arduous, this method can not identify a small number of *S. enterica *samples that lack either the O antigen alone or both the O and the H antigens [12]. Therefore there is a need for fast, sensitive and specific "in the field" detection, using nucleic acid-based technologies such as molecular beacon-based real-time PCR, to reduce the time needed to complete the assay, but also improve the level of accuracy and reliability.

In this study, molecular beacons [13-15] and real-time PCR technology are combined to develop a fast, sensitive, clear-cut method of detection of *Salmonella *spp. and its two most clinically significant serotypes from clinical, food and environmental specimens, which reduces the time taken to identify the *Salmonella *strain from an environmental sample, and is precise enough to distinguish between serovars. PCR-based methods targeting various genes are usually more rapid and sensitive than culture-based methods, and the high specificity and high sensitivity of molecular beacons means they can be successfully combined with real-time PCR assays, and so provide a quick, accurate method for detection and analysis, making them ideal for routine diagnosis. Recent studies show that real-time PCR is gradually replacing gel electrophoresis [16-24] as it is suitable for large numbers of samples and involves automatic and fast analysis, as well as being able to execute multiplex protocols. Also, like in all probe-based assays, molecular beacons offer additional specificity. Recent studies have employed molecular beacons in PCR for the detection of *Salmonella *[25-27].

Here the detection of *Salmonella *and the discrimination between *S*. Typhimurium and *S*. Enteritidis serotypes is done by targeting 133–136 nt regions of three genes, while an artificial internal amplification control (IAC) is also incorporated. The target for *Salmonella *spp is *invA*, as it is highly conserved in almost all *Salmonella *serotypes [28,29], and its specificity is apparent from its continuous use in previous studies [18,24,28,30-43]. The target for the specific detection of *S*. Enteritidis is *prot6E*, whose absence from *S*. Enteritidis strains appears to be very rare [18], and the *fliC *gene has been chosen as a single target for the presence of *S*. Typhimurium. The method described here for the detection of *Salmonella *spp. from environmental and food specimens, not only reduces the time taken to identify the *Salmonella *strain, but is also precise enough to distinguish between its clinically significant serovars.

## Methods

### Bacterial samples

The primary *Salmonella *samples used in this study (Table 1) were obtained from various animal, food and environmental sources at the Cyprus Veterinary Services (Ministry of Agriculture, Natural Resources and Environment, Nicosia, Cyprus), which is the National Reference Laboratory of *Salmonella *for Cyprus. The commercially available bacterial strains listed in Table 2 were obtained from the American Type Culture Collection (ATCC, Manasas, USA), the National Collection of Type Cultures (NCTC, Health Protection Agency, London, UK) and MERCK KGaA (Darmstadt, Germany). The reference *S. enterica *serovars listed in Table 3 were obtained from the Community Reference Laboratory for *Salmonella *at the National Institute for Public Health and the Environment (RIVM, Bilthoven, the Netherlands). Thirty-eight *S*. Typhimurium and *S*. Enteritidis samples as well as six different *Salmonella *serovars have been incorporated to ensure that the assay could correctly identify and differentiate between serotypes of *S. enterica*. Eighteen non-*Salmonella *bacterial samples were also included to ensure accurate experimental design and specificity to the *Salmonella *genus.

**Table 1:** Primary Bacterial Strains^*a*^

Bacterial strain	Sample ID	Source of Sample
*Salmonella *Enteritidis	CVS-140/1	Intestine from beef
*Salmonella *Enteritidis	CVS-141/1–5	Liver & ovaries from egg layer hens
*Salmonella *Enteritidis	CVS-4054/1	Lymph ganglions
*Salmonella *Enteritidis	CVS-4311/1	Intestine from canaries
*Salmonella *Enteritidis	CVS-4325/4, 5	Skin from neck of chicken
*Salmonella *Enteritidis	CVS-4421/1	Fish food
*Salmonella *Enteritidis	CVS-4516/1	Veal
*Salmonella *Enteritidis	CVS-4532/1	Parrot
*Salmonella *Enteritidis	CVS-4540/1	Parrot
*Salmonella *Enteritidis	CVS-4666/1	Faeces from egg layer hens
*Salmonella *Enteritidis	CVS-4756/1	Faeces from hens farmed for meat
*Salmonella *Enteritidis	CVS-4807//1–3	Skin from neck of chicken
*Salmonella *Enteritidis	CVS-4809/2	Skin from neck of chicken
*Salmonella *Enteritidis	CVS-4980/1	Faeces from chicken
*Salmonella *Enteritidis	CVS-5212/1	Faeces from egg layer hens
*Salmonella *Enteritidis	CVS-54/1	Faeces from egg layer hens
*Salmonella *Enteritidis	CVS-4792/1	Lymph ganglions
*Salmonella *Enteritidis	CVS-4754/1	Lymph ganglions
*Salmonella *Enteritidis	CVS-2553/4	Skin from neck of chicken
*Salmonella *Typhimurium	CVS-3225//1–5	Sheftalia (pork sausage)
*Salmonella *Typhimurium	CVS-4074/1	Parrot
*Salmonella *Typhimurium	CVS-4076/1	Pigeon
*Salmonella *Typhimurium	CVS-4255/1	Beef
*Salmonella *Typhimurium	CVS-4345/4, 5	Skin from neck of chicken
*Salmonella *Typhimurium	CVS-4979/1	Dust from egg layer hen cages
*Salmonella *Typhimurium	CVS-4981/1	Fish meal animal feed
*Salmonella *Typhimurium	CVS-5090/1	Faeces from finches
*Salmonella *Typhimurium	CVS-55/1	Faeces from egg layer hens
*Salmonella *Typhimurium	CVS-920/1–3	Egg yolk
*Salmonella *Typhimurium	CVS-131/2	Swab from swine
*Salmonella *Typhimurium	CVS-729/2	Swab from swine
*Salmonella *Typhimurium	CVS-3794/1	Water
*Salmonella *Typhimurium	CVS-3822/1	Water
*Salmonella *Typhimurium	CVS-1421/1	Lymph ganglions

^*a *^Identified by culture and serotyping methods as described in the Materials and Methods

**Table 2:** Commercially Available Strains

Bacterial Strains	Reference ID
*Salmonella *Typhimurium	14028^a^
*Salmonella *Enteritidis	13076^a^
*Staphylococcus aureus*	1803^b^
*Staphylococcus aureus*	25923^a^
*Bacillus cereus*	7464^b^
*Bacillus cereus*	11145^b^
*Bacillus cereus*	11778^a^
*Bacillus subtilis*	110649^c^
*Enterobacter aerogenes*	13048^a^
*Enterococcus faecalis*	29212^a^
*Escherichia coli*	25922^a^
*Escherichia coli O157*	35150^a^
*Listeria innocua*	11288^b^
*Listeria ivanovie*	11846^b^
*Listeria ivanovie*	19119^a^
*Listeria monocytogenes*	11994^b^
*Micrococcus luteus*	9341^a^
*Proteus vulgaris*	13315^a^
*Pseudomonas aeruginosa*	27853^a^
*Rhodococcus equi*	1621^b^

^a ^Strains obtained from American Type Culture

Collection (ATCC), Manassas, USA http://www.atcc.org

^b ^Strains obtained from National Collection of Type

Cultures (NCTC), London, UK http://www.nctc.org.uk

^c ^Strains obtained from MERCK KGaA, Darmstadt, Germany http://www.merck.de

**Table 3:** *Salmonella enterica *serovars^*a*^

Serovar	Reference ID
*Salmonella *Bredeney	1030/1
*Salmonella *Infantis	1030/4
*Salmonella *Anatum	1030/5
*Salmonella *Hadar	1030/6
*Salmonella *Newport	1030/7
*Salmonella *Typhimurium	1030/10
*Salmonella *Virchow	1030/11
*Salmonella *Enteritidis	1030/17

^*a *^Obtained from the Community Reference Laboratory for *Salmonella*, RIVM, Bilthoven, the Netherlands http://www.rivm.nl

### Bacterial cultures and serotyping

The detection of *Salmonella *spp. was performed based on the ISO 6579:2002 method. In brief, 25 g of clinical specimen (10 g in the case of minced meat in accordance with the EC regulation 2073/2005 – Microbiological Criteria for Foodstuffs) were added to 225 ml of buffered peptone water in a Stomacher^® ^bag, sealed and placed in a Stomacher^® ^blender for 3 min. The blended sample was incubated for 18 h at 37°C and a 0.1 ml aliquot of sample was inoculated into 10 ml Rappaport-Vassiliadis medium with Soya (RVS) and into 10 ml Muller-Kauffmann tetrathionate/novobiocin (MKTTn) broth; these cultures were incubated for 24 h at 41.5°C and 37°C, respectively. Each culture was inoculated into xylose lysine deoxycholate agar (XLD) and brilliant green agar (BGA) and incubated at 37°C for 24 h. One colony was selected from each XLD and BGA plate and spread onto nutrient agar for incubation at 37°C for 24 h. The resulting colonies were subject to biochemical analysis and serotyping. *Salmonella *spp. was characterised into different serovars on the basis of their surface (LPS, O-antigens) and flagellar antigens (H-antigens) as defined by the Kauffman-White Scheme [10,44] and based on the Global Salm-Surv laboratory protocol of the World Health Organisation (Global Salm-Surv, Serotyping of *Salmonella enterica *O and H antigen, Level 3 Training Course, WHO, 6^th ^edition, Jan. 2004).

To extract DNA for use in the molecular detection assay, bacteria were cultured on the XLD agar and one colony was selected and grown on nutrient agar. A colony was then selected and incubated in 5 ml nutrient broth, 1 ml of which was transferred into a 1.5 ml tube for centrifugation for 10 min at 18,000 rcf. The supernatant was discarded and the cell pellet was kept at -80°C until DNA extraction.

### Bacterial genomic DNA preparation

Bacterial genomic DNA was extracted from the cell pellets using QIAGEN DNeasy Blood and Tissue Kit (Hilden, Germany) according to the manufacturer's instructions. The purified DNA was eluted in 100 μl of AE buffer and the concentration was determined by measuring the optical density at 260 nm using a NanoDrop UV spectrophotometer (NanoDrop Technologies, USA). The extracted DNA was kept at -30°C until further use.

### Internal amplification control

An artificial 129 nt oligonucleotide fragment was designed as an IAC to be amplified by the same primers as the *invA *target. The IAC is a completely synthetic and unique oligonucleotide, designed to avoid sequence homology with any entries in the GenBank database, tested using the BLAST (Basic Local Alignment Search Tool) software [45].

### Primer and beacon design

All primers and molecular beacons described here were designed *de novo *for this study (See additional file 1: Oligonucleotide primers and molecular beacons in the real-time PCR assay). Small regions (133–136 nt) of the *invA*, *prot6E *and *fliC *genes were used as target sequences for the detection of *Salmonella *spp. *S*. Enteritidis and *S*. Typhimurium, respectively. The primers and the molecular beacons were designed based on sequences of the above genes found in the GenBank database http://www.ncbi.nlm.nih.gov/Genbank/index.html using BLAST [45]. The molecular beacons, target oligonucleotides and primers were synthesised by MWG-Biotech AG Ltd (Ebersberg, Germany) and the Midland Certified Reagent Company, Incorporated (Texas, USA). For the stem formation, the ends of each beacon were designed to have a high GC content and to be complementary to each other. All beacons were labelled with DABCYL, i.e., 4'-(4'-dimethylaminophenylazo) benzoic acid at the 3' end and with one of four fluorophores at the 5' end. Molecular beacon MBinvA, was labelled with the fluorophore FAM (Fluorescein); MBprot6E with TET (Tetrachloro-6'-carbofluorescein); MBfliC with HEX (hexachlorofluorescein); and MBIAC, with ROX (6'-carboxy-X-rhodamine). Within the loop element, each beacon contains a 22–25 nucleotide-long probe sequence complementary to the target. In addition to the probe sequence, each beacon has 4–5 of the 12 bases of its two arms also complementary to the target. In this non-traditional way, once the beacon is in the open structure, it binds more forcefully to the target and the hybrid formed is more stable, and the maximum distance possible between fluorophore and quencher is created.

### Thermal denaturation characteristics of the molecular beacons

To assess the thermodynamic characteristics, the quality and the purity of the molecular beacons used in this study, a melting curve analysis was performed on each using the 7900 HT Fast Real-Time PCR System (Applied Biosystems, Foster City, CA, USA). Briefly, the reaction consisted of a 25 μl solution containing 12.5 μl Platinum^® ^PCR Supermix (Invitrogen), 1 μl of the beacon probe at the appropriate concentration with or without 1 μl (100 pmol) of a single-stranded oligonucleotide perfectly complementary to the probe. The cycling parameters were as follows: 1 cycle for 2 min at 95°C followed by 50 cycles, each consisting of the data collection step for 30 s and a second step for 10 s, each starting at 80°C and employing auto-incrementation of -1°C per half-minute cycle until 31°C. Changes in fluorescence were measured at 490 nm and the data was collected at each temperature interval.

### PCR target standards synthesis, amplification and quantification

PCR target standards of the *fliC*, *invA*, *prot6E *and IAC target sequences were synthesised by PCR amplification using long overlapping primers. Each PCR reaction was performed in a 50-μl reaction volume containing 45 μl of Platinum^® ^PCR Supermix (Invitrogen, Inc., Carlsbad, CA, USA) and 25 pmoles each of the following primer pairs: for the *fliC *target, TFfliC and TRfliC; for the *invA *target, TFinvA and TRinvA; for the *prot6E *target, TFprot6E and TRprot6E; and for the IAC, TFIAC and TRIAC (See additional file 1: Oligonucleotide primers and molecular beacons in the real-time PCR assay). Amplification was performed with an activation step of 94°C for 30 s, followed by 20 cycles, each consisting of 94°C for 20 s, 68°C for 30 s and 72°C for 20 s, followed by a final extension step of 72°C for 5 min in an Eppendorf Mastercycler (Eppendorf AG, Hamburg, Germany). Three μl of the product from the first PCR was used in a secondary PCR in a 50-μl reaction volume containing 1 × of Platinum^® ^PCR Supermix (Invitrogen, Inc., Carlsbad, CA) and 20 pmoles each of the following PCR primer pairs: for the *fliC *target, 585 and 717; for the *invA *target, 302 and 437; for the *prot6E *target, 438 and 572; and for the IAC, 302 and 437 (See additional file 1: Oligonucleotide primers and molecular beacons in the real-time PCR assay). Amplification was performed with an activation step of 94°C for 30 s, followed by 40 cycles, each consisting of 94°C for 20 s, the annealing temperature for 30 s and 72°C for 20 s, followed by a final extension step of 72°C for 5 min in an Eppendorf Mastercycler (Eppendorf AG, Hamburg, Germany). The annealing temperature for the *fliC *primers was 59°C, for the *invA *primers 58°C, for the *prot6E *primers 56°C and for the IAC primers 58°C. The resulting product was then cleaned using the QIAquick PCR Purification Kit (QIAGEN GmbH, Hilden, Germany) and eluted in 50 μl of EB buffer. The PCR products were run on a 2% agarose gel with a 50 bp DNA ladder (Invitrogen) and the DNA concentration of each was measured on the NanoDrop ND-1000 UV Spectrophotometer (Wilmington, DE). The number of molecules per unit volume was calculated from the measured concentration and the molecular weight of each oligonucleotide. The amplified targets were then diluted to concentrations of 10^6^, 10^5^, 10^4^, 10^3^, 10^2 ^and 10 copies per 5 μl to be used as target standards of known concentration.

### Standard curves

Uniplex real-time PCR reactions were performed on 10-fold serial dilutions of the PCR targets, synthesised and prepared as described above. Reactions of 25 μl volume were set up, containing 12.5 μl Platinum^® ^qPCR Supermix-UDG (Invitrogen, Carlsbad, CA), 1 μl of forward primer and 1 μl of reverse primer (20 pmol/μl), 1 μl of the corresponding molecular beacon at the concentration determined appropriate from the melting curve analysis (4.9 pmol/μl MBinvA, 10 pmol/μl MBfliC, 4.4 pmol/μl MBprot6E and 50 pmol/μl MBIAC) 4.5 μl H_2_O and 5 μl of the PCR target standard. The reactions were performed on the 7900 HT Real-Time PCR System (Applied Biosystems, Foster City, CA, USA) and the cycling parameters were as follows: activation step at 95°C for 10 min, followed by 50 cycles, each consisting of 95°C for 15 s, 50°C for 30 s (data collection step) and 72°C for 30 s.

### Uniplex real-time PCR

The real-time PCR analysis was made with by the 7900 HT Fast Real-Time PCR System (Applied Biosystems) using the Platinum^® ^Quantitative PCR SuperMix-UDG (Invitrogen) on all of the samples described above. Each 25 μl uniplex PCR reaction contained 5 μl of the extracted DNA, and was carried out as described above. The fluorescence given out on hybridisation between each beacon and its target DNA was measured directly and the resulting amplification curves were processed immediately with the 7900 HT Sequence Detection Systems software v2.2.2 (Applied Biosystems, Foster City, CA). To verify that the fluorescence signals were due to PCR amplification of the template DNA and not any other contaminant, negative or non-template controls were also run, where sterile water replaced the DNA template in the reaction mixture.

### Double duplex real-time PCR

Having tested all sets of beacons and primers in uniplex reactions, the samples were run again in a two-step duplex assay. In step 1, 25 μl reactions were set up, containing 12.5 μl of Platinum Quantitative Supermix-UDG (Invitrogen), 1 μl of each of primers 302 and 437 (20 pmol/μl), 1 μl of MBIAC (50 pmol/μl), 1 μl of MBinvA (4.9 pmol/μl), 0.5 μl of the synthetic IAC (2 × 10^5 ^copies/μl). To this, 2 μl of 100-fold dilution of sample DNA were added and the volume was made up with sterile water or, in the case of non-template controls, the sample DNA was replaced with sterile water. In step 2, each reaction had a total volume of 25 μl consisting of 12.5 μl of Platinum Quantitative Supermix-UDG (Invitrogen), 1 μl of each of 572, 585 and 717 (20 pmol/μl), 1 μl of MBprot6E (4.4 pmol/μl) and 2 μl of MBfliC (10 pmol/μl). The final volume was reached by the addition of 2 μl of sample DNA and 3.5 μl of sterile water or, in the case of non-template negative control reactions, 5.5 μl of sterile water only. For both steps, PCR cycling conditions were as described for the standard curve analysis and uniplex reactions. The fluorescence given out on hybridisation between beacon and its target was measured at each cycle.

## Results

### Thermal denaturation characteristics of molecular beacons

**Figure 1:**
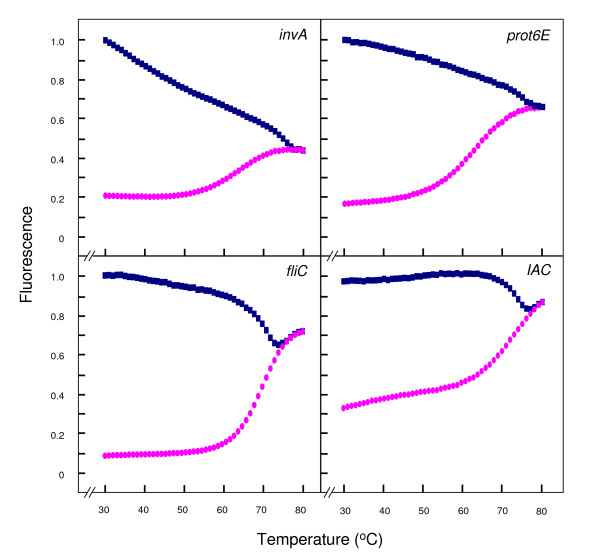
**Thermal denaturation profiles of the molecular beacons**. Thermal denaturation profiles of the molecular beacons used in this study as established by melting curve analysis (described in Materials and Methods). The figure shows normalised fluoresence thermal transitions of molecular beacon plotted in pink circles and beacon-target complexes plotted in blue squares.

Normalised fluorescence signals for both the beacon and the beacon-target hybrid were plotted against temperature to give a thermal denaturation profile for each beacon (Fig. 1). These profiles were created using an ABI 7900 HT Fast Real-Time PCR System (Applied Biosystems, Foster City, CA) to determine the optimal hybridisation temperature between the beacon and its target sequence. Perfectly complementary beacon-target hybrids exist at lower temperatures giving out a bright fluorescence signal. A progressive increase in temperature causes the hybrids to dissociate, followed by a marked decrease in fluorescence. Conversely, the beacons alone unravelled at high temperatures and exhibited a melting temperature above 60°C in all cases. In the temperature interval from 31 to 55°C, the probe-target hybrids elicited significantly stronger fluorescence than the probe alone, thus allowing the detection of target sequence at these temperatures. A temperature of 50°C was chosen as an optimal annealing temperature for subsequent real-time PCR studies. At this temperature the difference in fluorescence signal between beacon alone and beacon-target hybrids is large; in the absence of target any fluorescence detected is background level and the temperature is high enough to prevent less energetically favourable hybrids from forming, e.g., primer dimers or beacon-primer dimers. In the process of carrying out the melting curve analysis for all beacons, different concentrations were tested, to find the appropriate concentration at which the fluorescence signal was neither too low nor saturated. The concentrations at which the particular beacons exhibited the desired amount of fluorescence signal in these reactions were: MBIAC, 50 pmol/μl; MBinvA, 4.9 pmol/μl; MBprot6E, 4.4 pmol/μl; and MBfliC, 10 pmol/μl. Finally, these thermal denaturation profiles illustrate the good quality of the molecular beacons and their efficiency in hybridising with the appropriate target sequence.

### Standard curves and limit of detection

**Figure 2:**
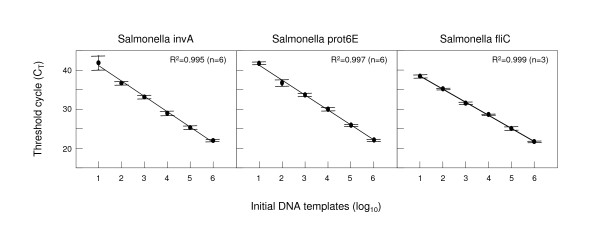
**Standard curves for targets *invA*, *fliC *and *prot6E***. Standard curves for molecular beacon-based real-time PCR detection of targets *invA*, *fliC *and *prot6E*. The plots illustrate the relationship of known number of target DNA copies per reaction to the threshold cycle of detection (C_T_) for each molecular beacon reaction. The C_T _is directly proportional to the log of the input copy equivalents, as demonstrated by the standard curves generated.

Standard curves were initially plotted to ensure the ability of each molecular beacon to detect its specific *Salmonella *target and the detection limit of the assay. The copy numbers of target standards used ranged from 10^1 ^to 10^6 ^copies per reaction. These plots represent how the amplification of DNA progresses with each log increase of target copy number. The small standard errors calculated from multiple values of the threshold cycle at which significant DNA amplification was observed (threshold cycle, C_T_) for each reaction and indicated on the graphs with horizontal lines above and below each plotted point, suggest that the PCR amplification is highly reproducible. The C_T _values for the target sequences depended on the initial DNA amount in each reaction as shown by the linear relationship of standard curves along a 6-log range which yields an R^2 ^correlation value higher than 0.994 in all three cases (Fig. 2). The correlation was 0.995 with 76% efficiency for *invA*, 0.997 and 84% efficiency for *prot6E *and 0.999 and 100% efficiency for *fliC*. As the reactions worked well for all target standard concentrations tested, the lower limit of detection for the assay was set to be 10 copies of the required target fragment per reaction. Based on the standard curves and the limit of detection of this assay, negative results were defined as those exhibiting C_T _values higher than 45.

### Detection of *S. enterica *alleles in bacterial samples by molecular beacon-based uniplex real-time PCR

The molecular beacon-based real-time PCR assay designed in this study was tested on environmental and food samples of *S*. Enteritidis and *S*. Typhimurium (Table 1), as well as several commercially available bacterial strains (Table 2) and various *Salmonella *serovars obtained from a reference laboratory for *Salmonella *(Table 3). All samples were investigated first by uniplex assays to detect *invA*, *prot6E *and *fliC *(Table 4). In the reaction for detection of *invA*, all 44 *Salmonella *samples were positive and all 18 non-*Salmonella *samples were undetectable. Positive results (≤ 10 copies of DNA per reaction) had C_T _values ranging from 15 to 25. In the *prot6E *reaction, all 21 *S*. Enteritidis samples gave positive PCR results and all 41 non-Enteritidis samples were negative. Positive samples for the *prot6E *gene had C_T _values ranging between 15 and 18 with one exception, the commercially available specimen of *S*. Enteritidis (Table 3) for which fluorescence detection significantly increased around cycle 30. Finally, in the *fliC *reaction, all 17 *S*. Typhimurium samples gave positive PCR results and all 45 non-Typhimurium samples were negative. Positive results had C_T _values ranging from 15 to 18 cycles. These results showed that the primers and beacons for each reaction work well individually and that they amplify and detect their target sequence with very high specificity and sensitivity. The C_T _values exhibited by the samples in these experiments, compared to the plot of the standards of known concentration, indicated that the extracted DNA from the bacterial samples was higher than the range of concentrations tested by the standards (>10^7 ^copies per reaction). Therefore 100-fold dilutions of all extracted DNA samples were prepared for use in the two-step duplex assay, so that the resulting C_T _values would fall within the range seen on the standard curves.

**Table 4:** Real-time PCR results obtained using the available bacterial strains

		PCR Result^*b*^			
		
Bacterial strain^*a*^	Reference ID	IAC^*c*^	*invA*	*prot6E*	*fliC*
*Salmonella *Enteritidis	CVS-140/1		+	+	-
*Salmonella *Enteritidis	CVS-141/1–5		+	+	-
*Salmonella *Enteritidis	CVS-4054/1		+	+	-
*Salmonella *Enteritidis	CVS-4311/1		+	+	-
*Salmonella *Enteritidis	CVS-4325/4, 5		+	+	-
*Salmonella *Enteritidis	CVS-4421/1		+	+	-
*Salmonella *Enteritidis	CVS-4516/1		+	+	-
*Salmonella *Enteritidis	CVS-4532/1		+	+	-
*Salmonella *Enteritidis	CVS-4540/1		+	+	-
*Salmonella *Enteritidis	CVS-4666/1		+	+	-
*Salmonella *Enteritidis	CVS-4756/1		+	+	-
*Salmonella *Enteritidis	CVS-4807//1–3		+	+	-
*Salmonella *Enteritidis	CVS-4809/2		+	+	-
*Salmonella *Enteritidis	CVS-4980/1		+	+	-
*Salmonella *Enteritidis	CVS-5212/1		+	+	-
*Salmonella *Enteritidis	CVS-54/1		+	+	-
*Salmonella *Enteritidis	CVS-4792/1		+	+	-
*Salmonella *Enteritidis	CVS-4754/1		+	+	-
*Salmonella *Enteritidis	CVS-2553/4		+	+	-
*Salmonella *Typhimurium	CVS-3225//1–5		+	-	+
*Salmonella *Typhimurium	CVS-4074/1		+	-	+
*Salmonella *Typhimurium	CVS-4076/1		+	-	+
*Salmonella *Typhimurium	CVS-4255/1		+	-	+
*Salmonella *Typhimurium	CVS-4345/4, 5		+	-	+
*Salmonella *Typhimurium	CVS-4979/1		+	-	+
*Salmonella *Typhimurium	CVS-4981/1		+	-	+
*Salmonella *Typhimurium	CVS-5090/1		+	-	+
*Salmonella *Typhimurium	CVS-55/1		+	-	+
*Salmonella *Typhimurium	CVS-920/1–3		+	-	+
*Salmonella *Typhimurium	CVS-131/2		+	-	+
*Salmonella *Typhimurium	CVS-729/2		+	-	+
*Salmonella *Typhimurium	CVS-3794/1		+	-	+
*Salmonella *Typhimurium	CVS-3822/1		+	-	+
*Salmonella *Typhimurium	CVS-1421/1		+	-	+
*Salmonella *Typhimurium	14028		+	-	+
*Salmonella *Enteritidis	13076		+	+	-
*Staphylococcus aureus*	1803		-	-	-
*Staphylococcus aureus*	25923		-	-	-
*Bacillus cereus*	7464		-	-	-
*Bacillus cereus*	11145		-	-	-
*Bacillus cereus*	11778		-	-	-
*Bacillus subtilis*	110649		-	-	-
*Enterobacter aerogenes*	13048		-	-	-
*Enterococcus faecalis*	29212		-	-	-
*Escherichia coli*	25922		-	-	-
*Escherichia coli O157*	35150		-	-	-
*Listeria innocua*	11288		-	-	-
*Listeria ivanovie*	11846		-	-	-
*Listeria ivanovie*	19119		-	-	-
*Listeria monocytogenes*	11994		-	-	-
*Micrococcus luteus*	9341		-	-	-
*Proteus vulgaris*	13315		-	-	-
*Pseudomonas aeruginosa*	27853		-	-	-
*Rhodococcus equi*	1621		-	-	-
*Salmonella *Bredeney	1030/1		+	-	-
*Salmonella *Infantis	1030/4		+	-	-
*Salmonella *Anatum	1030/5		+	-	-
*Salmonella *Hadar	1030/6		+	-	-
*Salmonella *Newport	1030/7		+	-	-
*Salmonella *Typhimurium	1030/10		+	-	+
*Salmonella *Virchow	1030/11		+	-	-
*Salmonella *Enteritidis	1030/17		+	+	-

^*a *^The origin of these samples has been described in tables 1, 2 and 3.

^*b *^Detectable real-time PCR amplification signal is denoted by the symbol (+) which indicates C_T _values ≤ 45. Undetectable signal (C_T _values >45) is denoted by (-) and indicates the absence of template DNA.

^*c *^IAC is an artificial internal amplification control that does not correspond to any of the submitted sequences in the Genbank.

### Detection of *S. enterica *alleles in bacterial samples by molecular beacon-based two-step duplex real-time PCR

**Figure 3:**
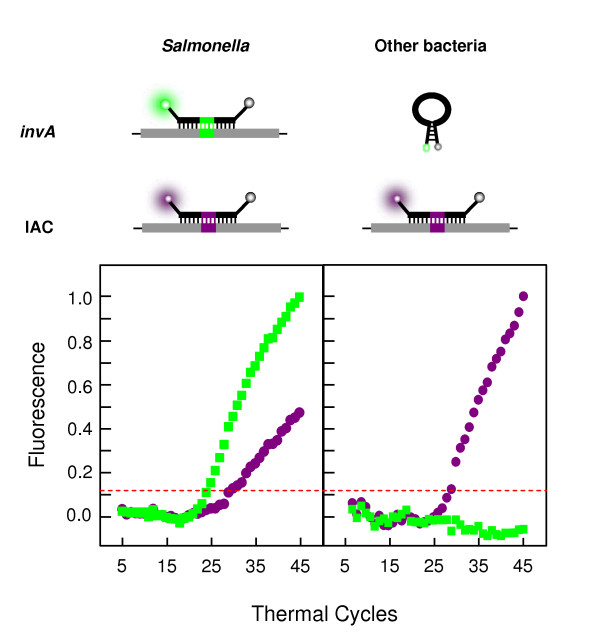
**Schematic real-time PCR results for the first step reaction**. Representative real-time PCR results as established by the first step multiplex reaction (described in Materials and Methods). The plots show average normalised linear amplification of representative samples shown for demonstration of typical results obtained from *Salmonella *and non-*Salmonella *bacteria. With DNA from non-*Salmonella *bacterial samples, only the IAC-specific, ROX-labelled molecular beacons hybridise to the IAC amplicons, generating violet fluorescence, whereas the *invA*-specific, FAM-labelled molecular beacons retain their stem-and-loop structure and cannot produce a green fluorescent signal. With DNA from *Salmonella *samples, both molecular beacons hybridise to their respective target amplicons and generate both green and violet fluorescence. The dashed line on the plots represents the normalised threshold for detection of fluorescence, the baseline above which fluorescence increases significantly on amplification and detection of the target sequence.

All samples were subsequently tested employing a double duplex strategy, where the first step of the assay involved a real-time PCR reaction containing the primers and beacons for the amplification and detection of both the *invA *gene and the IAC, a quantity of which was inserted into each reaction. This step of the assay determined the presence or absence of the *invA *gene to verify whether the sample was *Salmonella *or not. Simultaneous amplification and detection of the IAC in all samples ensured accurate PCR performance, therefore excluding the possibility of false-negative results. Amplification and detection of both *invA *and the IAC were clear in all *Salmonella *samples, whereas only the IAC amplification was detected in non-*Salmonella *samples. Representative amplification plots from *Salmonella *and other bacteria for the first step reaction are seen in Fig. 3. The results demonstrate that this reaction correctly recognises samples in which *Salmonella *exist from samples in which it does not.

**Figure 4:**
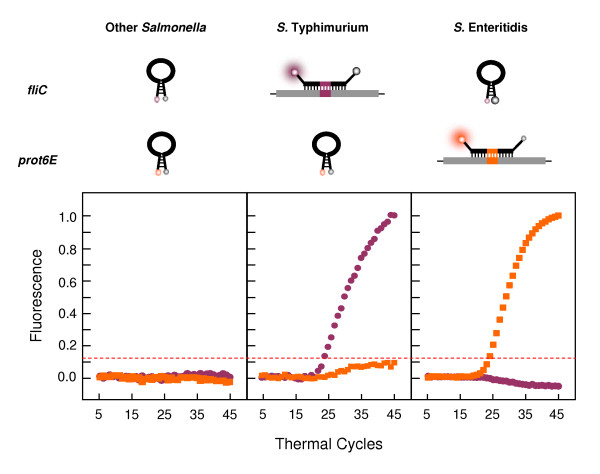
**Schematic real-time PCR results for the second step reaction**. Representative real-time PCR results as established by the second step multiplex reaction (described in Materials and Methods). The plots show average normalised linear amplification of representative samples shown for demonstration of typical results obtained from *S*. Typhimurium, *S*. Enteritidis and other *Salmonella *samples. With DNA from *S*. Typhimurium strains, only *fliC*-specific, HEX-labelled molecular beacons hybridise to the amplicons, generating pink fluorescence, whereas the *prot6E*-specific, TET-labelled molecular beacons retain their stem-and-loop structure and cannot produce an orange fluorescent signal. With DNA from *S*. Enteritidis strains, the *prot6E*-specific, TET-labelled molecular beacons hybridise to their target amplicons and produce an orange fluorescent signal, whereas the *fliC*-specific, HEX-labelled molecular beacons remain dark. With DNA from other *Salmonella *serotypes, no target amplicons are detected and both molecular beacons remain dark. The dashed line on the plots represents the normalised threshold for detection of fluorescence, the baseline above which fluorescence increases significantly on amplification and detection of the target sequence.

All samples found positive for *invA *in the first step were then tested in the second step of the assay, another duplex real-time PCR reaction containing the components for amplification and detection of both *prot6E *and *fliC *targets. In all *S*. Typhimurium samples *fliC *was the only target detected, in all *S*. Enteritidis samples *prot6E *was the only target detected and in all other *Salmonella *samples, both targets were undetected. The results show that this reaction clearly and accurately distinguishes between *S*. Typhimurium strains, *S*. Enteritidis strains and other *Salmonella *serotypes. Representative amplification plots from *S*. Typhimurium, *S*. Enteritidis and other *Salmonellae *for the second step reaction are seen in Fig. 4, clearly showing that the *prot6E *and *fliC *components designed in this study work well together in a multiplex real-time PCR reaction.

In both the uniplex and double duplex assays, non-template controls were included to verify the absence of false-positive results. In all cases they exhibited undetectable amplification of the targets (C_T _>45).

### Selectivity of the real-time assay

The selectivity and accuracy of the test is measured by calculating the values for specificity and sensitivity. Specificity is the probability that the PCR will be negative among specimens that should not possess the gene and is calculated using the formula: true negative/(true negative + false positive). Sensitivity shows the strength of the test in recognising what we are looking for, i.e., in correctly identifying the specific serotype. The formula used for estimation of sensitivity is: true positive/(true positive + false negative). For the reaction targeting the *invA *gene, all 44 *Salmonella *samples investigated were positive indicating a sensitivity of 100%. The specificity was also 100% since all non-*Salmonella *samples gave negative results, with undetectable fluorescence signals after 50 cycles. In the *prot6E *reaction all *S*. Enteritidis samples analysed were identified correctly with positive PCR results and all non-Enteritidis samples were negative for this target. Thus, this reaction also had sensitivity and specificity of 100%. Similarly, in the assay for *fliC *detection, all *S*. Typhimurium samples tested were positive for the target. The assay's sensitivity was 100%, matched by an equal specificity value as all non-Typhimurium samples tested gave negative PCR results.

## Discussion

Traditional serotyping of *S. enterica *is based on the detection of certain antigens using microbiological techniques and culturing, which are time-consuming and laborious. This study exploits real-time PCR, molecular beacons and genetic variation between different serotypes to devise a quick, accurate and simple assay to reliably identify a bacterial sample as *Salmonella enterica *and further distinguish it as serotypes *S*. Typhimurium or *S*. Enteritidis, the two serovars most commonly associated with food-borne gastroenteritis. The assay described in this study can analyse a large number of samples very quickly, and can also identify as few as 10 copies of target DNA per reaction, potentially even in the presence of thousands of copies of other serotypes. The short completion time of this assay and the ability of PCR to detect even dead bacterial cells, even though these are no longer infectious, highlight the drawbacks of the culturing method and the need to start using modern molecular techniques as opposed to conventional microbiology methods where applicable. PCR sensitivity is superior to that of the bacteriological culturing methods, as it can detect *Salmonellas *with atypical biochemical features, reducing false-negative results, and it will not mistakenly detect non-*Salmonella *bacteria, reducing the chances of false-positive data [27]. However, further research is necessary to ensure that molecular assays alone can efficiently detect *Salmonella *spp. and its serotypes.

A variety of bacterial samples were used to test the specificity of the assay in the detection of the genus *Salmonella*. At the same time a number of *Salmonella *strains were included to ensure that the detection tests for serovars *S*. Typhimurium and *S*. Enteritidis were specific. The study includes strains from clinical and environmental samples as well as commercially available strains, and a significant number of *S*. Typhimurium and *S*. Enteritidis samples as well as other *Salmonella *serotypes and non-*Salmonella *bacteria. This broad range of samples was included to test the efficacy of the assay. Three genes were specifically targeted: the *invA *gene specific to the genus *Salmonella; *the *prot6E *gene specific to *S*. Enteritidis; and the *fliC *gene specific to *S*. Typhimurium. Due to its specificity, the *invA *gene is an excellent potential target for the detection of *S. enterica *strains alone [18,24,28,30-43]. The fact that it is unique for *S. enterica *and rarely absent from it [46], ensures high specificity and sensitivity in detection assays. The *prot6E *gene is located on a highly conserved, low copy number, 60-kb virulence plasmid specific to *S*. Enteritidis and its absence appears to be very rare [18]. Finally, the *fliC *gene codes for the H1 antigen of *Salmonella*. Targeting the *fliC-i *allele greatly increases the specificity for *S*. Typhimurium identification. In order to detect *S*. Typhimurium with the highest specificity, three genes could ideally be targeted, coding for antigens O:4, H1:i and H2:1,2, as it is the only serovar with this antigen combination [47]. However, this would not only raise the costs of the assay but would compromise the simplicity of design and the potential for including further molecular beacons in the multiplex reaction for identification of other target serotypes. Thus, in this study, as in some other studies [48,49], the *fliC *gene has been chosen as a single target for the presence of *S*. Typhimurium. By designing the *fliC *beacon using a *S*. Typhimurium sequence from the GenBank database as a template, the assay exhibits high sensitivity. However, although it performed with 100% specificity with this particular set of samples, in a different set of samples, e.g., with other *S. enterica *serotypes like Kentucky that also possess the H1:i antigen, it might yield different results. Many different genes have been targeted in previous studies [16,22,25,26,30,47,48,50-54]. However, the above targets did not prove to be specific enough for unique detection and identification.

The IAC used is a synthetic and unique oligonucleotide designed *de novo *for this study. The fact that this IAC is co-amplified with the *invA *fragment using the same primer set but detected by a distinct beacon, does not appear to alter the precision and accuracy of the real-time PCR, and quantification of original target DNA is still possible even in the presence of the control. However, the standard curve protocol for *invA *in the presence of the IAC should be performed for a correct quantitative approach if the assay is to be used for quantification. The *invA *gene has been used as an internal amplification control in other studies [18], but its application is limited to *Salmonella *assays alone. Furthermore, it has been found that in some rare cases, this gene may be absent and is therefore unreliable as an amplification control even for studies incorporating *Salmonella *specimens alone. This IAC sequence matches no organism in the NCBI libraries and could potentially be used in any such detection assays.

The assays for the *invA*, *fliC *and *prot6E *genes all had a sensitivity and specificity score of 100%. All 45 *Salmonella *samples were positive, with 100% sensitivity. Positive results (>10 copies of DNA per reaction) had C_T _values ranging from 15 to 25. One exception, the commercially available specimen of *S*. Enteritidis (Table 3), had a C_T _value of approximately 30. Since the *prot6E *gene is located on a virulence plasmid, its absence would not be surprising. Plasmid profiling should be performed to explain the unusually high C_T _value observed for this specific specimen. This raises the question of whether selecting a target on a plasmid is a wise choice, but this absence of this plasmid has been found to be rare from *S*. Enteritidis and the low copy numbers (1–2) of the plasmid in the cells make possible the conversion of the assay to a quantitative one which would be correct to a factor of 2. Therefore, using this target for quantification would depend on the accuracy required.

Our study is the first to incorporate four molecular beacons with real-time PCR in a double duplex PCR protocol to detect *Salmonella *spp., *Salmonella *Enteritidis and *Salmonella *Typhimurium in a single assay. Strong fluorescence signals were observed in all positive PCR results in both the uniplex and the duplex assays, indicating the efficiency of the design in the primers and beacons. The sensitivity and specificity of the design and procedure described here give the assay the potential to be converted into a quantitative method, directly applied to samples without the requirement of pre-enrichment stages, making use of the standard curves.

## Conclusion

This molecular beacon-based real-time PCR assay is extremely useful for any laboratory in possession of a real-time PCR. It is a fast, reproducible, simple, specific and sensitive way to detect nucleic acids, which could be used in clinical diagnostic tests in the future. The design of the assay gives it potential to be used for quantification, for detection of multiple other serotypes of *Salmonella *or to be modified for the detection of other bacterial samples. Also, more significantly, the sensitivity of the test and its confirmed low limit of detection, are promising factors for the important switch to direct detection from real clinical and environmental samples which have not been previously cultured and have low numbers of bacteria.

## Authors' contributions

AVH participated in the assay design, sample preparation, real-time PCR experimental procedures, the analysis and interpretation of the results and drafted the manuscript. VLD carried out sample preparation, real-time experimental procedures, analysis and interpretation of results and drafted the manuscript. MAE carried out the bacterial culturing and serotyping techniques, sample selection, bacterial pellets isolation and helped with the manuscript preparation. CKK participated in sample selection and donated samples for this study. LGK conceived and designed the assay, coordinated the study and participated in sample selection and analysis and interpretation of results. All authors read and approved the final manuscript.

## Supplementary Material

Additional File 1 **Oligonucleotide primers and molecular beacons in the real-time PCR assay**. Table of primer and molecular beacon sequences used in this study. Click here for file
