# Supplementary material for: Molecular beacon-based real-time PCR detection of primary isolates of Salmonella Typhimurium and Salmonella Enteritidis in environmental and clinical samples
Source: BMC Microbiol. 2009 May 19;9:97. doi: 10.1186/1471-2180-9-97 (PMC2689230; doi:10.1186/1471-2180-9-97)
Supplement: Additional File 1 — Oligonucleotide primers and molecular beacons in the real-time PCR assay. Table of primer and molecular beacon sequences used in this study. [file 1471-2180-9-97-S1.doc]

Additional File 1. Oligonucleotide primers and molecular beacons in the real-time PCR assay

| Designation *^a^* | Target  Gene | Sequence | Position *^b^* | Amplicon  Length (nts) | Gene Accession Number | Reference |
| --- | --- | --- | --- | --- | --- | --- |
|  |  |  |  |  |  |  |
| **PCR Primers** |  |  |  |  |  |  |
| 302 (F) | *invA* | TTGGCGATAGCCTGGCGGTG | 302-321 | 136 | DQ644615 | This study |
| 437 (R) | *invA* | TGTTTACCGGGCATACCATCCAGAG | 413-437 |  | DQ644615 | This study |
| 438 (F) | *prot6E* | ggcaccgcagcaatggttgg | 438-457 | 135 | U66901 | This study |
| 572 (R) | *prot6E* | GGTCGAGCTACAGAGAGTCACAC | 550-572 |  | U66901 | This study |
| 585 (F) | *fliC* | taacacctgctgctgtcaatgcgg | 904-881 | 133 | AY649721 | This study |
| 717 (R)  **Target Primers *^c^***  TFinvA (F)  TRinvA (R)  TFprot6E (F)  TRprot6E (R)  TFfliC (F)  TRfliC (R)  TFIAC (F) *^d^*  TRIAC (R) | *fliC*  *invA*  *invA*  *prot6E*  *prot6E*  *fliC*  *fliC* | ACTCTTGCTGGCGGTGCGACTT  TTGGCGATAGCCTGGCGGTGGGTTTTGTTGTCTTCTCTATTGTCACCGT  GGTCCAGTTTATCGTTATTACCAAAGGTTCAGAACGTGTCGCGGAAGT  CGCGGCCCGATTTT  TGTTTACCGGGCATACCATCCAGAGAAAATCGGGCCGCGACTTCCGCG  ACACGTTCTGAACCTTTGGTAATAACGATAAACTGGACCACGGTGACA  ATAGAGAAGACAACAAAACC  GGCACCGCAGCAATGGTTGGGTTCGGGGGAGACTATACCTACAGGGG  CACAATAACCGTAACCGGAGAGGCGCTCATCGGTCCTGCTGTAGATGC  AAGGGTGCCTAAGGTT  GGTCGAGCTACAGAGAGTCACACTAACCTTAGGCACCCTTGCATCTAC  AGCAGGACCGATGAGCGCCTCTCCGGTTACGGTTATTGTGCCCCTGTA  GGTATAGTCTCCCCCGAAC  TAACACCTGCTGCTGTCAATGCGGCTTTAGCCTCTGTCAAATCAGCAT  TTGCAACTTGTACATTTTTCACATCCTCAGTTGCTGTCGCAGGTAGTCC  ACCTGTAAGCGGGG  ACTCTTGCTGGCGGTGCGACTTCCCCGCTTACAGGTGGACTACCTGCG  ACAGCAACTGAGGATGTGAAAAATGTACAAGTTGCAAATGCTGATTT  GACAGAGGCTAAAG  TTGGCGATAGCCTGGCGGTGGCTGTATCGACGATGATCTGCTACTAGC  TCGAGGGAGCCTCTGCTGAGTAGCGACACTGATCGCCCTCGACTAGCT  CGGTACAT  TGTTTACCGGGCATACCATCCAGAGATGTACCGAGCTAGTCGAGGGCG  ATCAGTGTCGCTACTCAGCAGAGGCTCCCTCGAGCTAGTAGCAGATCA  TCGTCGATACAGC | 793-772  302-412  322-437  438-548  458-572  904-794  880-772  N/A  N/A | 136  135  133  129 | AY649721  DQ644615  DQ644615  U66901  U66901  AY649721  AY649721  N/A  N/A | This study  This study  This study  This study  This study  This study  This study  This study  This study |
|  |  | Continued |  |  |  |  |
| **Molecular Beacons** *^e^* |  |  |  |  |  |  |
| MBinvA | *invA* | FAM-GGTCGCGCCGCGACTTCCGCGACACGTTGCGACC-DABCYL | 383-404 |  | DQ644615 | This study |
| MBprot6E | *prot6E* | TET-CGTCGCCAGCAGGACCGATGAGCGCCTCGCGACG-DABCYL | 504-525 |  | U66901 | This study |
| MBfliC | *fliC* | HEX-GCACGCGGACTACCTGCGACAGCAACTGAGGGCGTGC-DABCYL | 832-808 |  | AY649721 | This study |
| MBIAC *^d^* |  | ROX-CGAGCCGCTACTCAGCAGAGGCTCCCTCGGGCTCG-DABCYL | N/A |  | N/A | This study |

*^a^* PCR primer, oligonucleotide and molecular beacon names as these appear in the text; Orientation of the PCR primer is indicated in parenthesis: F, forward; R, reverse

*^b^* Positions correspond to the appropriate Genebank sequences. In the case of molecular beacons, the positions correspond to the target recognition sequences of the molecular beacon only. In the case of the *fliC* positions, the beacon and primer design is based on the reverse complement of the gene sequence.

*^c^* Each genetic target sequence was generated by PCR using forward and reverse overlapping oligonucleotides spanning the entire sequence as described in the Materials and Methods.

*^d^* IAC denotes an artificial internal amplification control, which does not correspond to any of the submitted sequences in the Genebank. IAC target primers amplify the IAC target DNA and MBIAC is a molecular beacon which recognizes the IAC.

*^e^* Underlined regions denote the sequences that form the stem of the molecular beacons; FAM denotes fluorescein; TET, Tetrachloro-6′-carbofluorescein; HEX, hexachlorofluorescein; ROX, 6′-carboxy-X-rhodamine, and DABCYL, 4′-(4′-dimethylaminophenylazo) benzoic acid.
